# Laryngeal Cancer Survival in Northern United Kingdom: A Multi‐Centre Retrospective Observational Study

**DOI:** 10.1111/coa.70138

**Published:** 2026-07-08

**Authors:** Amar Rajgor, Rhona Hurley, Catriona M. Douglas, Claire Paterson, James Moor, Shane Lester, Sara Sionis, Katherine Davies, James O’Hara, Gareth Inman, Terry Jones, David Winston Hamilton, Northern Head & Neck Alliance, Amarkumar Rajgor, Rhona Hurley, Catriona M. Douglas, Claire Paterson, James Moor, Shane Lester, Sara Sionis, Katherine Davies, James O'Hara, Gareth Inman, Terry Jones, David Winston Hamilton, Sarah Hill, Seamus O'Neill, Alison McLoughlin, Jemy Jose, Winson Wong, Michael Ho, Debbie Horne, Jarrod Homer, Matthew Kennedy, Emma Kinloch, Robert Metcalf, Iain Varley, Thomas Carroll, Sarah Healy, Helen Cocks, Michael Nugent, Leo Vassilou, Panos Kyzas, David Conway, John Greenman, Andrew Schache, Jason Fleming, Joanne Patterson, Keith Hunter, Paula Parvulescu, Rachel Brooker, Richard Shaw, Stephanie Meysner, Ola Rominiyi, Olena Mandrik

**Affiliations:** ^1^ National Institute for Health & Care Research Doctoral Fellow in Otolaryngology, Population Health Sciences Institute, Newcastle University Newcastle‐Upon‐Tyne UK; ^2^ Newcastle Upon Tyne Hospitals NHS Foundation Trust, Freeman Hospital Newcastle‐Upon‐Tyne UK; ^3^ NHS Greater Glasgow & Clyde Newcastle‐Upon‐Tyne UK; ^4^ Glasgow Head and Neck Research (GLAHNC) Group Glasgow UK; ^5^ CRUK Scotland Institute and School of Cancer Sciences, College of Medical and Veterinary Sciences, University of Glasgow Glasgow UK; ^6^ Leeds Teaching Hospitals NHS Foundation Trust Leeds UK; ^7^ South Tees Hospitals NHS Foundation Trust Middlesbrough UK; ^8^ Sheffield Teaching Hospitals NHS Foundation Trust Sheffield UK; ^9^ Liverpool University Hospitals NHS Foundation Trust Liverpool UK; ^10^ Liverpool Head & Neck Centre Liverpool UK

**Keywords:** (chemo)radiotherapy, head and neck surgery, larynx cancer, oncology, outcomes

## Abstract

**Objectives:**

Laryngeal cancer incidence has risen globally, yet survival outcomes have shown limited improvement over recent decades. This study evaluated contemporary outcomes across multiple centres in Northern UK and examined stage‐ and treatment‐specific survival.

**Design:**

Multi‐centre retrospective observational study.

**Setting and Participants:**

Data were collected for 2028 patients with biopsy‐proven squamous cell carcinoma of the larynx treated with curative intent between 2015 and 2021 across six tertiary head and neck cancer centres (Newcastle, Glasgow, Liverpool, Sheffield, Leeds, and Middlesbrough).

**Main Outcome Measures:**

Overall survival (OS) and disease‐specific survival (DSS) were estimated using Kaplan–Meier analysis and compared using log‐rank testing.

**Results:**

Median follow‐up was 40 months (mean age 66.8 years; 81% male). Overall mortality was 41% and disease‐specific mortality 20%. Five‐year OS declined with advancing stage: 72.0% (T1), 54.5% (T2), 50.6% (T3), and 45.2% (T4). In unadjusted analyses, surgery in T1 disease was associated with higher survival (5‐year DSS 93.6% vs. 88.5%), while in advanced disease chemoradiotherapy was associated with the highest DSS (5‐year DSS 70.2%), although differences were not statistically significant. No significant differences in DSS were observed across year of diagnosis (2015–2021).

**Conclusions:**

Survival outcomes for laryngeal cancer remain poor and have shown limited improvement over time. These findings provide important real‐world insights into contemporary outcomes and highlight the need for earlier detection, prevention strategies, and prospective collaborative research to improve future outcomes.

## Key Points

- Largest UK collaborative study of laryngeal cancer outcomes, including 2028 patients treated across six specialist centres.
- Five‐year survival remains low (57.1%), with no significant improvement seen between 2015 and 2021.
- Earlier diagnosis and precision medicine are essential to improve future outcomes for patients with laryngeal cancer.

## 1 Introduction

Since the 1990s, the incidence of head and neck cancer has risen by 33%, making it the seventh most common cancer worldwide [1]. Among these cases, a significant proportion originate in the larynx, affecting approximately 2400 individuals in the United Kingdom (UK) each year [2]. Two landmark randomised controlled trials (RCTs) in the New England Journal of Medicine over 25 years ago heralded a shift towards chemo‐radiotherapy to improve oncological outcomes [3, 4]. However, despite the global rise in laryngeal cancer incidence, mortality and recurrence rates remain static or, at their worst, show deterioration [5, 6]. Furthermore, laryngeal cancer results in enduring health challenges, including difficulties with breathing, speech and swallowing. These challenges are a consequence of both the disease and the life‐changing interventions.

Recent advancements in personalised medicine, driven by genomics, molecular profiling, radiomics, and immunotherapy, have transformed cancer treatment [7]. However, laryngeal cancer has yet to benefit significantly from these innovations, with no tissue biomarker available [8]. The Northern Head & Neck Alliance (NHNA), established in 2022 under the Northern Health Science Alliance (NHSA), aims to address this gap by uniting expertise and resources from nine major centres in Northern UK to advance head and neck oncology.

## 2 Objective

This collaborative multi‐centre study evaluates outcomes of laryngeal cancer in a contemporary population marked by high rates of comorbidities and harmful risk factors like smoking and alcohol use. It represents one of the largest investigations into survival patterns of laryngeal cancer in the UK, offering deeper insights into prognosis.

## 3 Methods

### 3.1 Patient Population

Data spanning 6 years (January 2015 to December 2021) were collected retrospectively from six major head and neck specialist centres in Northern UK. Following assessment with the UK Health Research Authority decision tool, this project did not require formal research ethics approval. All participating centres contributed anonymised retrospective data. These included NHS Greater Glasgow & Clyde, Leeds Teaching Hospitals, Liverpool University Hospitals, Newcastle Upon Tyne NHS, Sheffield Teaching Hospital, and South Tees Hospitals. Inclusion criteria comprised patients diagnosed with biopsy‐proven squamous cell laryngeal carcinoma managed with curative intent (*n* = 2028).

Data collection at each participating centre was undertaken by clinicians within the Northern Head & Neck Alliance collaborative, including consultants and registrars, using a standardised data collection proforma. This collaborative approach ensured consistency in data capture across sites. All contributors are acknowledged within the Northern Head & Neck Alliance authorship and collaborator group. Data was subsequently collated centrally into an Excel database for analysis. Collected data included age, gender, smoking status at diagnosis, tumour subsite, AJCC 8th edition tumour (T), node (N) and metastasis (M) stage, primary treatment, overall mortality, and disease‐specific mortality. All cases were staged according to the AJCC 8th edition criteria. For patients diagnosed before the introduction of AJCC 8th edition, information was retrospectively reclassified where possible to ensure consistency. Both clinical and pathological staging data were used, depending on availability at each centre. Early‐stage disease encompassed T1–T2, while advanced disease referred to T3–T4.

Patients lacking sufficient follow‐up data were excluded from survival analyses (*n* = 9). For included patients, cases with missing data for individual variables (e.g., smoking status, cause of death) were retained in the analysis, with missingness reported in Table 1.

**TABLE 1:** Patient characteristics of cohort.

Regional larynx data summary	
Patients included	
Total (*n*)	2028
NHS Greater Glasgow & Clyde	670 (33%)
Leeds Teaching Hospitals NHS Trust	257 (13%)
South Tees Hospitals NHS Trust	256 (13%)
Newcastle Upon Tyne NHS Trust	220 (11%)
Sheffield Teaching Hospital NHS Trust	451 (22%)
Liverpool University Hospitals NHS Trust	174 (9%)
Baseline demographics	
Age^a^	
Age (mean ± SD)	66.8 ± 10.8
Gender^a^	
Male	1660 (81%)
Female	342 (19%)
Smoking status^b^	
Non‐smoker	92 (9.2%)
Ex‐smoker	375 (37%)
Current smoker	536 (53%)
Tumour subsite	
Subglottis	33 (2%)
Glottis	985 (49%)
Supraglottic	624 (31%)
Transglottic	233 (12%)
Not defined	153 (8%)
AJCC TNM staging 8th edition	
T‐stage	
T1	650 (32%)
T2	431 (21%)
T3	575 (28%)
T4	372 (18%)
N‐stage	
N0	1604 (79%)
N1	126 (6%)
N2	267 (13%)
N3	31 (2%)
M‐stage	
M0	2018 (99.5%)
M1	10 (0.5%)
Primary treatment	
Surgical	952 (47%)
Non‐surgical	1075 (53%)
Specific modality	
Radiotherapy	838 (41%)
Chemoradiotherapy	237 (12%)
Surgery alone	786 (39%)
Surgery with adj. therapy	167 (8%)
Follow up data	
Median follow up (range)	40 months (1–161)
Overall mortality	
Alive	1202 (59%)
Deceased	826 (41%)
Disease‐specific mortality^c^	
Alive	1202 (62%)
Disease‐associated death	384 (20%)
Death unrelated to disease	342 (18%)

*Note:* Baseline characteristics of cohort, encompassing demographics.

Abbreviation: TNM stage = treatment modality and survival rates.

^a^ *n* = 26.

^b^ *n* = 1025.

^c^ *n* = 100.

### 3.2 Treatment Approaches

Treatment modality was classified according to the initial curative‐intent therapy. Comparisons were made between three main groups: surgery (with or without adjuvant therapy), primary radiotherapy, and primary chemoradiotherapy. Patients who later underwent salvage treatments were also analysed under their initial treatment category. Care was led by multidisciplinary teams and aligned with national guidance.

### 3.3 Statistical Analysis

Statistical analyses were conducted using SPSS version 29.0.1.1. Kaplan–Meier survival analyses assessed survival, including overall survival (OS) and disease‐specific survival (DSS) rates. Comparisons between groups were made using the log‐rank test, with statistical significance defined as *p* < 0.05. Cause of death was determined from hospital records and death certificates. Patients were censored at the date of last known follow‐up if alive or lost to follow‐up.

## 4 Results

### 4.1 Patient Characteristics

The study cohort comprised 2028 patients who underwent curative management for laryngeal cancer. The mean age of the patients was 66.8 years, with a notable male predominance (81%). The median follow‐up time was 40 months. Most cases occurred in the glottis (49%). Early‐stage disease (T1‐2) was observed in 53% of patients (*n* = 1081), while 46% (*n* = 947) had advanced‐stage disease (T3‐4). The overall mortality rate was 41%. Further detail is shown in Table 1.

### 4.2 Survival Outcomes

In this cohort, the estimated 2‐year and 5‐year overall survival (OS) rates for all stages and sites were 80.3% and 57.1% respectively (Figure 1A). Disease‐specific survival (DSS) rates for the same intervals were 85.5% and 76.0% respectively (Figure 1B). When analysed by year of diagnosis, disease‐specific survival remained unchanged between 2015 and 2021, with no significant differences between years (log‐rank *p* = ns; Figure S1).

**FIGURE 1:**
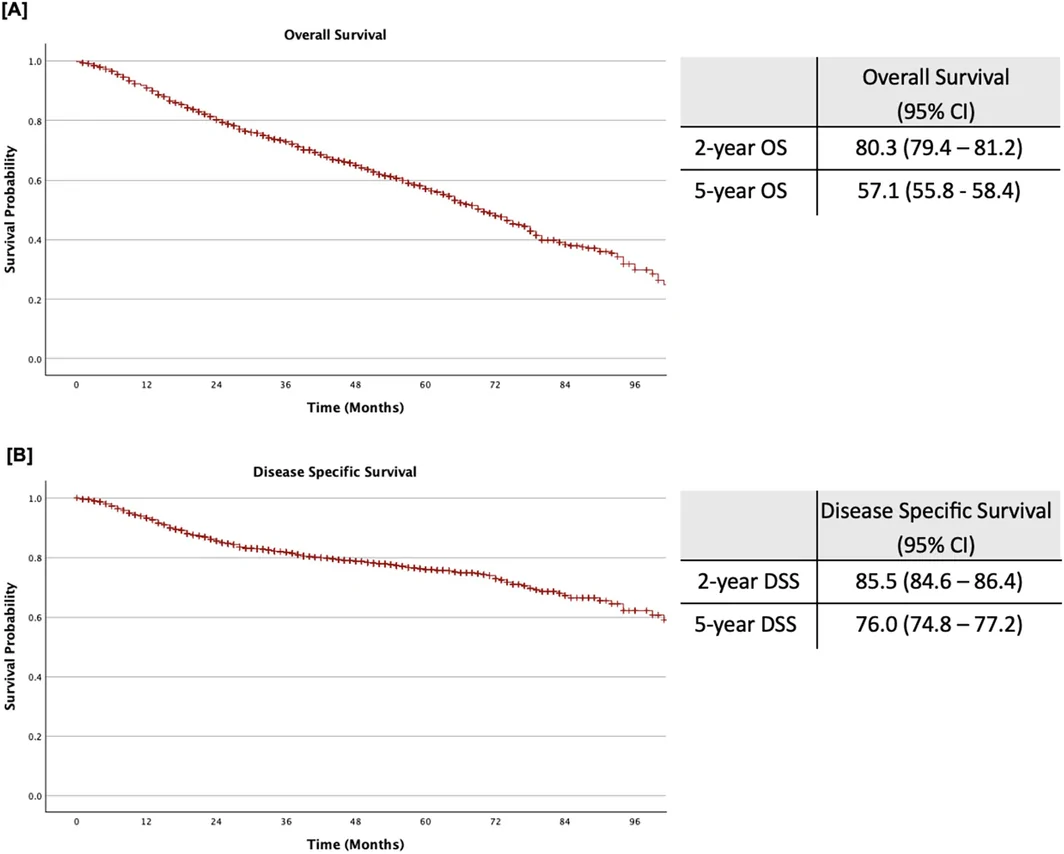
Kaplan Meier survival curves with associated estimated survival rates for entire cohort. [A] Overall survival: Kaplan Meier survival curve with associated estimated overall survival rates for entire cohort. [B] Disease specific survival: Kaplan Meier survival curve with associated estimated disease‐specific survival rates for entire cohort.

The cohort were further stratified by AJCC Tumour Stage (T1–T4) (Figure 2). As depicted in Figure 2A, the estimated 5‐year OS rates of T1, T2, T3, and T4 disease were 72.0%, 54.5%, 50.6%, and 45.2% respectively. Whilst the 5‐year DSS rates for T1, T2, T3, and T4 disease were 91.6%, 77.3%, 68.7%, and 59.7% (Figure 2B). For early (T1–T2) and advanced (T3–T4) laryngeal cancer cohorts, the estimated 5‐year disease‐specific survival (DSS) rates were 85.7% and 65.1%, respectively. These differences in survival across tumour stages were statistically significant on log‐rank testing (*p* < 0.01).

**FIGURE 2:**
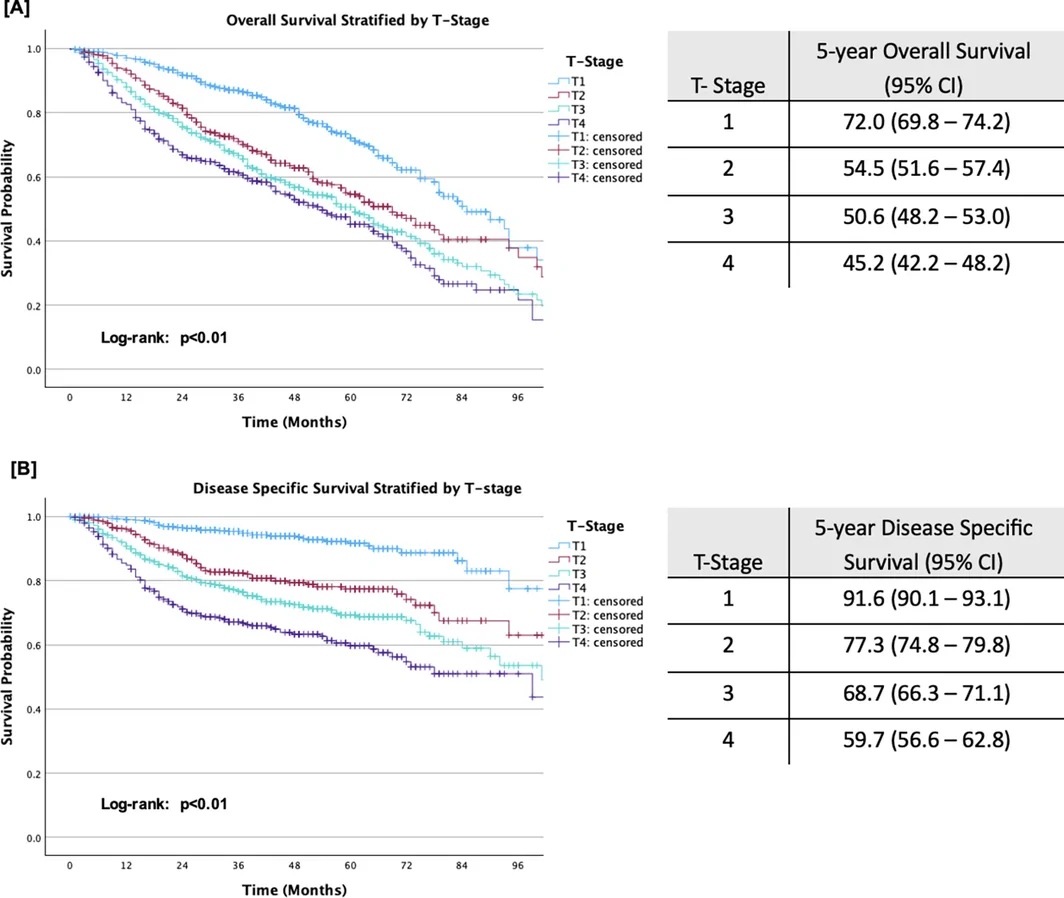
Kaplan Meier survival curves with associated survival rates for patients with laryngeal cancer stratified by Tumour Stage (T‐Stage). [A] Overall survival: Kaplan Meier survival curve with associated estimated overall survival rates for entire cohort stratified by T‐Stage. [B] Disease specific survival: Kaplan Meier survival curve with associated estimated disease‐specific survival rates for entire cohort stratified by T‐Stage Log‐rank *p* values are displayed on the figure.

As demonstrated in Figure 3, treatment outcomes differed according to tumour stage. For T1 disease, surgical management was associated with higher disease‐specific survival compared with non‐surgical treatment (5‐year DSS 93.6% vs. 88.5%, log‐rank *p* = 0.03) (Figure 3A). However, this represents an unadjusted analysis and should be interpreted with caution, as differences may reflect underlying patient selection rather than treatment effect (Figure 3A). In contrast, for T2 disease there was no statistically significant difference between surgical and non‐surgical treatment (5‐year DSS 84.7% vs. 75.6%, log‐rank *p* = 0.39) (Figure 3B). In locally advanced disease (T3–T4), patients undergoing chemoradiotherapy had the highest 5‐year DSS (70.2%), followed by radiotherapy (64.4%) and surgery (64.2%), although differences were not statistically significant (log‐rank *p* = 0.11) (Figure 3C).

**FIGURE 3:**
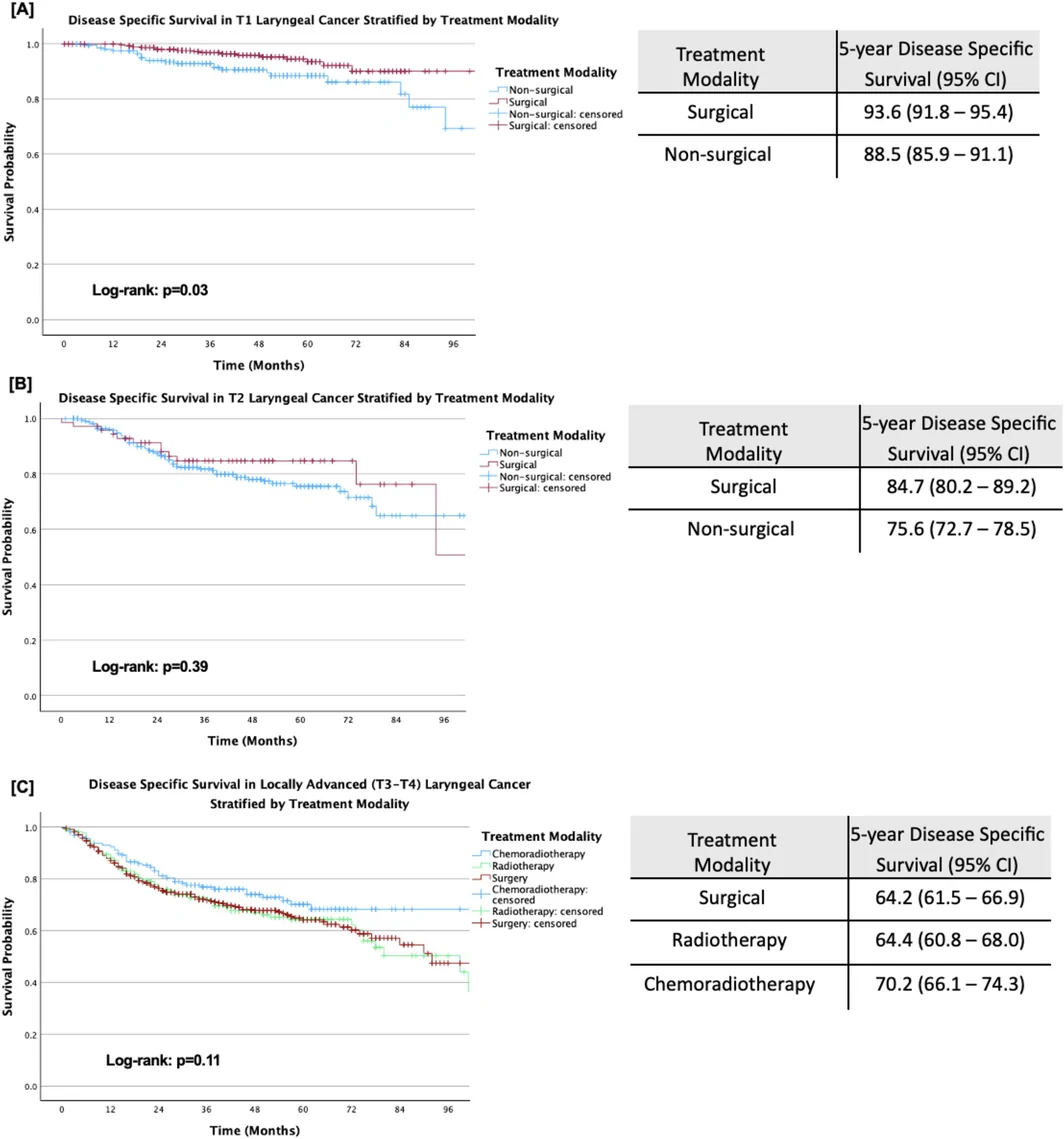
Kaplan–Meier survival curves with associated survival rates for patients with laryngeal cancer stratified by Treatment Modality. [A] Disease‐specific survival in T1 laryngeal cancer: Kaplan–Meier survival curve with associated estimated disease‐specific survival rates stratified by treatment modality (surgical vs. non‐surgical). [B] Disease‐specific survival in T2 laryngeal cancer: Kaplan–Meier survival curve with associated estimated disease‐specific survival rates stratified by treatment modality (surgical vs. non‐surgical). [C] Disease‐specific survival in locally advanced laryngeal cancer (T3–T4): Kaplan–Meier survival curve with associated estimated disease‐specific survival rates stratified by treatment modality (surgery, radiotherapy, chemoradiotherapy). Log‐rank *p* values are displayed on the figure.

## 5 Discussion

To date, this is one of the largest collaborative studies investigating laryngeal cancer outcomes in the UK. The findings reveal a 5‐year OS rate of 57.1% among all patients undergoing curative management in the Northern UK region. The estimated 5‐year OS rates for different stages of disease—T1, T2, T3, and T4—were 72.0%, 54.5%, 50.6%, and 45.2%, respectively. These results underscore the bleak survival prospects for this patient population. Poor survival figures also reflect the multiple comorbidities and harmful risk factors common in this group. Our results also demonstrate superior outcomes with surgery in T1 disease, consistent with prior reports favouring surgical management in this group [6]. Although this finding should be interpreted cautiously given the unadjusted nature of the analysis. In T2 disease there was no statistically significant difference between surgical and non‐surgical treatment, reflecting clinical equipoise and potential heterogeneity in patient selection. In advanced disease, chemoradiotherapy achieved the most favourable survival, though differences between modalities did not reach statistical significance.

These treatment comparisons should be interpreted with appropriate caution. The survival analyses are based on unadjusted Kaplan–Meier estimates and do not account for potentially important confounding variables such as age, comorbidity burden, performance status, or more granular tumour characteristics (e.g., T1a vs. T1b disease). As this is not a randomised study, treatment allocation is influenced by clinical decision‐making, and differences in outcomes may therefore reflect underlying patient or disease factors rather than treatment effect alone. While more detailed adjustment or stratified analyses would be valuable, these were not feasible within the current dataset due to incomplete and heterogeneous availability of key variables across centres. Importantly, this reflects the realities of large‐scale, multi‐centre retrospective data. Nonetheless, the strength of this study lies in its size and real‐world nature, providing a contemporary overview of treatment patterns and outcomes across the UK. As such, these findings are best interpreted as highlighting patterns of care and areas of clinical equipoise, rather than establishing definitive treatment superiority, and they provide an important foundation for future prospective and more granular analyses.

Nocini et al. documented a global rise in laryngeal cancer incidence and prevalence over the last 30 years, with rates increasing by 12% and 24%, respectively. Notably, Europe has the highest incidence (5.45 cases per 100 000) and mortality (2.57 per 100 000). Despite these alarming trends, survival rates have either stagnated or worsened during this period [5]. United States studies report 5‐year OS rates for all stages ranging from 54.26% in 2004 to 55.8% in 2011. Similarly, another study found 5‐year OS and DSS rates of 63% and 80% in 2001, indicating a decline from previous decades [5]. Even in 1995, the best‐reported 5‐year OS rate was 63% [6]. Unlike progress in other cancers, survival in laryngeal cancer shows little improvement, reflecting limited therapeutic advances. Consistent with these international trends, our contemporary dataset (2015–2021) demonstrated no significant differences in disease‐specific survival between diagnosis years (Figure S1), reinforcing that survival outcomes have remained static in recent years. Attempts to identify superior treatments have produced conflicting results. In addition to the limited progress in therapeutic innovation, worsening survival rates may also be attributable to broader contextual factors. Supportive interventions such as smoking cessation, alcohol reduction, nutritional optimisation, and speech and swallowing rehabilitation have the potential to enhance both survival and quality of life. These aspects were not included in our analysis but remain important considerations for improving outcomes in this patient group. The impact of the COVID‐19 pandemic must also be considered, as treatment delays, particularly for radiotherapy and chemoradiotherapy, together with exacerbation of socioeconomic disparities, are likely to have contributed to poorer outcomes. Future improvements in laryngeal cancer care will require not only advances in oncological treatment but also earlier diagnosis through public awareness, systematic reduction of modifiable risk factors, and the implementation of prehabilitation programmes to optimise patients for treatment.

In our cohort, patients with T2 disease exhibited a 5‐year OS and DSS rate of 54.5% and 77.3%, respectively. A potential reason for this could be the misclassification of T3 disease as T2. Misclassification may occur because distinguishing between T2 and T3 disease is subjective, relying on characteristics like immobile vocal cords or paraglottic tissue involvement in imaging studies. This distinction is crucial as T2 tumours typically undergo single modality treatment while T3 tumours receive dual modality. An evolving argument suggests treating a subset of T2 patients, specifically those with impaired vocal cord mobility, with dual modality treatment, as research indicates their outcomes resemble T3 disease [9]. Another promising avenue is artificial intelligence (AI). For instance, Liu et al. developed a nomogram based on radiomic analysis of CT images, which can differentiate between T2 and T3 disease [9]. While promising, true improvement requires larger‐scale, multi‐centre studies incorporating ‘multi‐omics’ research. ‘Multi‐omics’ analyses biological data types such as genomics, proteomics, radiomics, and metabolomics, holding significant potential in laryngeal cancer [7].

Emerging AI technologies like radiomics offer opportunities for earlier detection, improved staging, and innovative treatments [7]. These approaches provide insights into tumour behaviour and its microenvironment but to achieve real‐world benefit they require robust external validation and integration with clinical and molecular datasets through prospective multi‐centre trials [10]. Without such steps, AI‐driven and multi‐omics approaches risk remaining exploratory rather than practise‐changing. Potential prognostic markers under investigation include patient‐related factors (e.g., tobacco use, muscle mass) and tumour‐related markers (e.g., IDO, ‘immunoscore’, PDL‐1). Integrating these markers within a unified multiomics framework could allow tailored treatments based on disease biology, moving away from a one size fits all approach towards precision medicine. Prospective databases collecting comprehensive data, such as pathological samples, clinical information, imaging datasets, and patient‐reported outcomes (e.g., the ‘LARyngeal Cancer CoHort’ or LARCH), will be central to this strategy [8].

This study provides one of the largest evaluations of laryngeal cancer outcomes in the UK, incorporating data from six major head and neck cancer centres across the North of England and Scotland. As such, it offers a comprehensive, real‐world depiction of outcomes in contemporary practise. Our findings highlight the importance of addressing wider determinants of survival. These include earlier diagnosis through improved awareness, better access to timely treatment, reduction of risk factors such as smoking and alcohol use, and maintaining patient engagement with long‐term follow‐up. The stagnation in survival therefore reflects not only the biological challenges of laryngeal cancer but also broader public health and health system issues. Collaborative research will be essential, but parallel investment in prevention, early detection, and patient support is equally critical.

Limitations include the retrospective design, missing data for some variables, and the use of unadjusted survival analyses based on Kaplan–Meier estimates. As such, the analyses do not account for potentially important confounding factors, including comorbidity burden, performance status, and more detailed tumour characteristics. The study did not assess comorbidity impacts, treatment‐related morbidity, or recurrence, as these data were not consistently available across centres. In addition, details of specific surgical techniques (e.g., endoscopic vs open partial vs total laryngectomy) were not consistently available and could not be stratified. As a multicentre retrospective study, there are inherent challenges in ensuring data consistency and completeness, and variations in clinical practise and documentation across centres may introduce heterogeneity. While the size and real‐world nature of this cohort are key strengths, comparisons between treatment modalities should not be interpreted as definitive evidence of superiority, as the underlying patient populations differ in clinically meaningful ways. Nonetheless, the study provides important contemporary insights into outcomes in this population and highlights the need for prospective, standardised datasets to enable more granular and adjusted analyses in the future.

## 6 Conclusions

This collaborative study underscores the stagnation in laryngeal cancer survival and research progress. The findings emphasise the need to shift focus from determining treatment superiority to embracing precision medicine for defining treatment pathways in this disease. Earlier detection, effective prevention strategies, and novel therapies will be crucial. Collaborative efforts such as those coordinated by the NHNA may help drive meaningful advancements in understanding and managing laryngeal cancer.

## Author Contributions

A.R., D.W.H., and T.J., with contributions from J.O.H., designed the study. The core writing committee and key data collators consisted of A.R., R.H., C.M.D., C.P., J.M., S.L., S.S., K.D., J.O.H., G.I., T.J., and D.W.H. All authors, including collaborators across the NHNA, reviewed and provided critical revisions to the manuscript.

## Funding

There were no specific grants from any funding agency, commercial or not‐for‐profit sectors. Of note, AR is currently a National Institute for Health and Care Research (NIHR) Doctoral Fellow (Award Reference: NIHR302984). The views expressed in this publication are those of the authors and not necessarily those of the NIHR. RH is supported by a Cancer Research UK funded clinical academic training program (TRACC) PhD studentship and a Cancer Research UK award to GI (A29802).

## Ethics Statement

No ethical approval was required after evaluation using the Health Research Authority decision tool (available at: http://www.hra‐decisiontools.org.uk/research/).

## Consent

The authors have nothing to report.

## Conflicts of Interest

The authors declare no conflicts of interest.

## Supporting information


**Figure S1:** Kaplan–Meier disease‐specific survival curves by year of diagnosis.

## Data Availability

The data that support the findings of this study are available on request from the corresponding author. The data are not publicly available due to privacy or ethical restrictions.

## Appendix A

**Authorship Statement & Participation (Northern Head & Neck Alliance) Writing committee & key data collators:** Amarkumar Rajgor, Rhona Hurley, Catriona M. Douglas, Claire Paterson, James Moor, Shane Lester, Sara Sionis, Katherine Davies, James O'Hara, Gareth Inman, Terry Jones, David Winston Hamilton.

**Project Management:** Sarah Hill (The NHSA—Project Manager), Seamus O'Neill (The NHSA—Chief Executive).

**Additional Collaborators Across Northern Head & Neck Alliance:** Alison McLoughlin (East Lancashire Hospitals NHS FT), Jemy Jose, Winson Wong (Hull University Teaching Hospitals NHS FT), Michael Ho (Leeds Teaching Hospitals NHS FT), Debbie Horne (Liverpool Head & Neck Cancer Centre); (Liverpool University Hospitals NHS FT), Jarrod Homer (Manchester University NHS FT), Matthew Kennedy (Newcastle upon Tyne Hospitals NHS Foundation Trust (NUTH)), Emma Kinloch, Robert Metcalf (Salivary Gland Cancer UK), Iain Varley (Sheffield Childrens Hospital NHS FT), Thomas Carroll (Sheffield Teaching Hospitals NHS FT), Sarah Healy (South Tees Hospitals NHS FT), Helen Cocks, Michael Nugent (Sunderland & South Tyneside NHS FT), Leo Vassilou, Panos Kyzas (University of Central Lancashire (UCLan)), David Conway (University of Glasgow), John Greenman (University of Hull), Andrew Schache, Jason Fleming, Joanne Patterson, Keith Hunter, Paula Parvulescu, Rachel Brooker, Richard Shaw, Stephanie Meysner (University of Liverpool & Liverpool University Hospitals NHS FT), Ola Rominiyi, Olena Mandrik (University of Sheffield).
