# Supplementary material for: Laryngeal Cancer Survival in Northern United Kingdom: A Multi‐Centre Retrospective Observational Study
Source: Clin Otolaryngol. 2026 Jul 8;51(6):867–74. doi: 10.1111/coa.70138 (PMC13631802; doi:10.1111/coa.70138)
Supplement: Supplementary file 1 [file COA-51-867-s001.docx]

**Log-rank: p=0.38**

**Supplementary Figure 1: Kaplan–Meier Disease-Specific Survival Curves by Year of Diagnosis**

Kaplan–Meier survival curves with associated disease-specific survival rates for patients with laryngeal cancer, stratified by year of diagnosis (2015–2021). No significant differences in disease-specific survival were observed across years (*log-rank p = ns*).
